# Research on a lead-acid battery fault detection method based on LSTM-AE-Cosine

**DOI:** 10.1371/journal.pone.0357237

**Published:** 2026-09-15

**Authors:** Le Liu, Hongsheng Xu, Hai Tang, Jianing Cui, Li Feng, Ke Deng

**Affiliations:** 1 School of Intelligent Connected Vehicle, Hubei University of Automotive Technology, Shiyan, China; 2 Shiyan Key Laboratory of Air-Ground Crowd Cooperation Technology and Application (Hubei University of Automotive Technology), Hubei University of Automotive Technology, Shiyan, China; Commonwealth Scientific and Industrial Research Organisation, AUSTRALIA

## Abstract

Existing fault detection methods for lead-acid batteries in commercial vehicles are subject to several critical limitations, such as low detection accuracy and a shortage of labeled fault data. To address these issues, this paper presents an unsupervised deep learning framework for anomaly detection. In the framework, a hybrid LSTM-Autoencoder architecture is used to integrate the temporal feature extraction capability of long short-term memory networks with the reconstruction mechanism of Autoencoders, thus markedly improving the temporal modeling of battery data. Since the detection performance is highly dependent on the selection of reconstruction error metrics, multiple such metrics are employed in the model, and are systematically evaluated and compared to determine the most suitable metric for battery anomaly detection. The model is trained separately for static and driving conditions based on corresponding subsets of the battery datasets. With a fixed model structure and experimental conditions, model performance is evaluated using various reconstruction error metrics, and Bayesian optimization is used to identify the optimal detection threshold for each metric. Experimental results show that detection performance changes with different reconstruction error metrics and cosine similarity yields the best performance. This study offers a practical solution for online battery diagnosis and helps lower vehicle maintenance costs.

## Introduction

Lead-acid batteries are essential to vehicle electrical systems, supporting engine starting, onboard electrical equipment, and stable system operation [1]. In commercial vehicles, battery failures increase warranty replacement costs and disrupt vehicle availability. Early detection is therefore important, but lead-acid batteries often show only subtle degradation before failure, making periodic inspection and post-failure replacement insufficient [2]. Real-world vehicle data further complicate detection because fault samples are scarce and operational records often lack fine-grained fault labels.

Traditional density-based and classical machine learning methods remain limited for this setting. Density-based methods are sensitive to parameter selection, while classical machine learning methods rely on handcrafted features and have limited ability to represent high-dimensional, nonlinear, and non-stationary battery time series. Commercial vehicle battery signals contain noise, operating-state transitions, and temporal dependencies caused by driving behavior, charging, parking, load changes, and battery aging. These properties make it difficult for traditional methods to separate abnormal degradation from normal operating variation.

Reconstruction-based deep anomaly detection offers a practical alternative because autoencoder models can learn normal temporal patterns from abundant normal data. However, detection performance depends not only on the model architecture but also on the reconstruction error metric used to quantify deviations. Point-wise errors such as MSE, MAE, RMSE, and Euclidean distance mainly measure amplitude differences, which may confuse benign voltage fluctuations with faults. Shape- and trend-aware metrics can provide complementary anomaly evidence by capturing global temporal consistency.

This paper proposes the Long Short-Term Memory Autoencoder (LSTM-AE) model, with the following specific work:

1. The model is built on an autoencoder that includes an encoder, decoder, and repeat vector layer. This structure allows the model to learn normal behavior patterns from time-series battery data without relying on labeled anomalies.
2. Long Short-Term Memory (LSTM) units are used in both the encoder and decoder to capture long-term dependencies in battery operational data. This enhances the model’s ability to represent the global temporal correlations inherent in sequential battery signals.
3. To enhance the sensitivity of the model to time-series data, we introduce multiple reconstruction error evaluation metrics to conduct comparative experiments, thereby identifying the optimal reconstruction error metric for lead-acid battery fault detection scenarios.
4. Bayesian optimization is applied to automatically select the optimal anomaly detection threshold, improving both detection accuracy and efficiency.

## Background

With the development of Internet of Vehicles technology, commercial vehicle battery data are no longer limited to laboratory measurements, but can be continuously collected under real operating conditions. This change makes it necessary to reconsider lead-acid battery fault detection from the perspective of real-world time-series data.

### Fault detection for commercial vehicle lead-acid batteries

Lead-acid batteries are essential components in commercial vehicle electrical systems. They support engine starting, supply power to onboard electrical equipment, and help maintain stable operation of the vehicle electrical system. Battery failure directly affects vehicle availability and increases maintenance and after-sales service costs. Therefore, early fault detection is important for identifying abnormal battery degradation before it develops into vehicle starting failure or power-supply instability.

Unlike laboratory battery tests, commercial vehicle battery monitoring relies on real-world telemetry collected during daily operation. Battery voltage changes with engine starting, alternator charging, parking, driving load, ambient temperature, vehicle usage patterns, and battery aging. These factors introduce noise, state transitions, and non-stationary patterns into voltage sequences. As a result, abnormal battery behavior is difficult to separate from normal operating variation.

Fault detection is further limited by the scarcity of labeled fault samples. In practical vehicle operation, normal battery records are much easier to collect than early-fault records, and manual labeling requires maintenance records or expert inspection. This makes supervised fault classification difficult to apply at scale. Traditional methods that rely on fixed thresholds, handcrafted rules, or manually selected features are therefore less suitable for commercial vehicle battery telemetry, where operating conditions vary across vehicles and driving scenarios.

### Unsupervised time-series fault detection

Battery monitoring data are continuous time-series records collected during vehicle operation. Compared with isolated measurements, voltage sequences contain temporal dependencies that reflect charging, discharging, parking, driving, and degradation processes. Battery faults usually appear as abnormal temporal patterns rather than single isolated outliers [3]. For example, early degradation may change the voltage trend, fluctuation pattern, or state-transition behavior over a period of time. This makes time-series anomaly detection suitable for identifying abnormal battery behavior from real-world telemetry. In practical commercial vehicle operation, normal battery data are abundant, whereas labeled fault samples are limited and difficult to obtain. Manual labeling often depends on maintenance records, expert inspection, or manufacturer fault reports, which makes large-scale supervised learning difficult. Unsupervised anomaly detection is therefore more suitable for this task because it can learn normal battery behavior without requiring large numbers of labeled fault samples. Autoencoder-based models provide an effective framework for this setting. An autoencoder learns compact representations of normal time-series patterns and reconstructs the input sequence from these representations. When an abnormal sequence is given to a model trained mainly on normal data, the reconstruction discrepancy usually increases. This reconstruction discrepancy can then be converted into an anomaly score for fault detection. However, the final anomaly score depends strongly on the reconstruction error metric. Point-wise metrics mainly measure amplitude differences, while Cosine similarity focuses on whether the reconstructed sequence preserves the overall voltage trend. This motivates the use of reconstruction-based unsupervised anomaly detection for lead-acid battery fault detection, where early faults may appear as contextual or collective deviations in voltage sequences.

## Related work

Lead-acid battery fault detection in vehicle time-series data is a challenging task, and many studies have investigated battery anomaly detection because this information is critical for reliable vehicle operation and early maintenance decision-making.

### Battery fault detection methods

Traditional battery fault detection mainly includes rule-based, feature-based, and classical machine learning methods. Rule-based and feature-based studies use expert indicators, discharge-curve features, impedance parameters, or statistical distributions to identify abnormal battery behavior [4]. Sun et al. [5] diagnosed lead-acid battery degradation from voltage and current indicators. Murari et al. [6] identified failing automotive lead-acid batteries through discharge-curve correlation features, while Hariprakash et al. [7] used impedance-related parameters for online health monitoring. These methods provide interpretable diagnostic evidence, but their features depend on known fault mechanisms and relatively stable operating conditions.

Classical machine learning methods reduce the dependence on fixed thresholds by learning abnormal patterns from data, and local-density generative anomaly detectors extend this data-driven direction [8]. Qiu et al. [9] applied local outlier factor to battery energy-storage fault diagnosis, and Syed et al. [10] used shape-based clustering for battery anomaly detection in data centers. Shan et al. [11] and Yang et al. [12] developed DBSCAN-based strategies for voltage inconsistency and multi-scenario fault diagnosis. Isolation-forest and sliding-window methods have also been used for abnormal voltage detection and early warning [13–16]. However, many traditional methods still rely on handcrafted statistics, distance assumptions, or short-window samples, while comparative evaluations show that unsupervised detector performance depends on data structure and parameter calibration [17]. This limits their ability to describe long-term temporal evolution in commercial vehicle lead-acid battery telemetry.

### Reconstruction-based deep fault detection

Reconstruction-based methods address the scarcity of labeled battery fault samples by learning normal patterns and detecting samples with large reconstruction errors. Deep feature learning has also been used to extract representations for anomaly detection [18]. Sakurada and Yairi [19] demonstrated autoencoder-based anomaly detection, and later surveys summarize the broad use of autoencoder variants in time-series and battery diagnostics [20,21]. LSTM-AE models extend this idea by adding temporal memory. They have been applied to indoor air-quality monitoring [22] and electric-motor anomaly detection [23], showing their ability to capture sequential dependence. Other variants, including SVD-AE, federated AE, and metric-learning AE models, further improve representation learning in multivariate anomaly detection tasks [24–26]. Generative and application-specific deep-learning studies further show the broader use of representation learning in anomaly-related modeling tasks [27–30].

Although AE and LSTM-AE models can learn compact representations, their detection results still depend strongly on how reconstruction error is defined. Many studies emphasize model architecture while using default point-wise errors such as MSE, MAE, RMSE, or Euclidean distance. These errors measure magnitude differences at aligned time points, but they may ignore whether the reconstructed sequence preserves the global trend. For real vehicle battery data, normal operating changes can shift voltage magnitude because of charging state, load, temperature, and driving behavior. A magnitude-only reconstruction error may therefore confuse operating variation with early degradation.

### Reconstruction error metrics

Reconstruction error metrics convert the difference between the input sequence and its reconstruction into anomaly scores, thereby determining which type of deviation becomes visible to the detector. Point-wise metrics, such as Mean Squared Error, Mean Absolute Error, Root Mean Squared Error, and Euclidean distance, emphasize amplitude differences at corresponding time points. In vehicle battery data, however, normal operating patterns may contain noise and slight temporal shifts, so shape- and alignment-aware metrics can provide complementary information by capturing trend consistency and temporal morphology. Lin et al. [31] showed that anomaly-detection performance and false alarms can change with scoring and calibration choices. Kwak et al. [32] used Cosine similarity to capture pattern consistency in time-series anomaly detection, while Gao et al. [33] used Fast Dynamic Time Warping to compare sequences under temporal misalignment. These studies suggest that magnitude, trend, and alignment describe different anomaly evidence.Unlike prior studies that primarily focus on model architecture, this study compares multiple reconstruction error metrics within the same LSTM-AE framework and identifies Cosine similarity as an effective anomaly score for lead-acid battery fault detection.

## Related theories and technologies

This study requires a technical basis that can represent battery voltage sequences, learn normal operating behavior, and quantify deviations from reconstructed patterns. Therefore, the section introduces the theories and technologies needed to support the proposed LSTM-AE-Cosine method.

### Autoencoder

**Fig 1:**
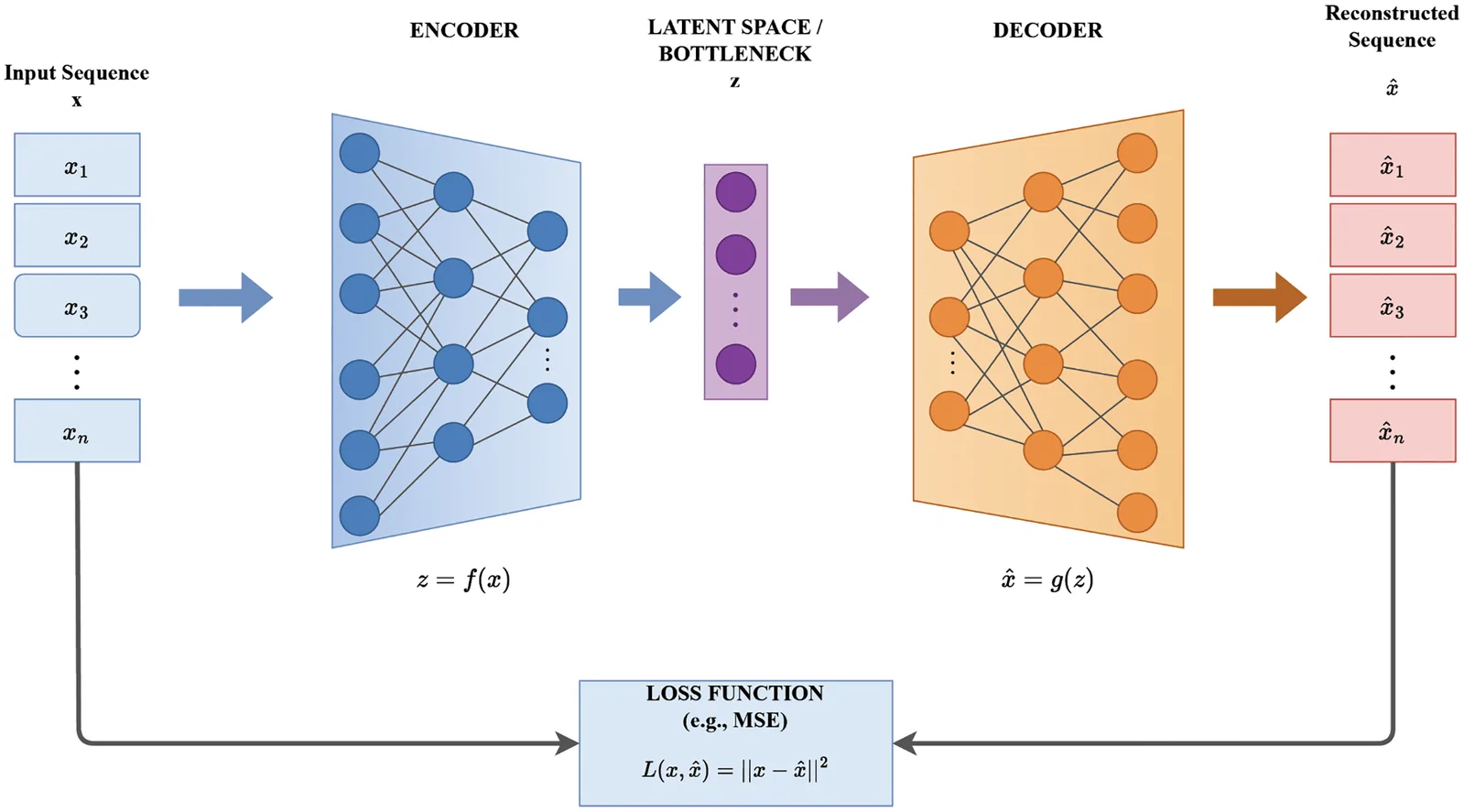
Structure of the autoencoder.


Autoencoders are unsupervised neural networks that learn compact representations by mapping high-dimensional inputs into a low-dimensional latent space and reconstructing them back to the original space [34]. As illustrated in Fig 1, the model consists of an encoder and a decoder, enabling nonlinear feature extraction through an unsupervised learning paradigm. Given an input sample X, the encoder projects it into a latent representation:

$$\begin{array}{c} h=f_{\theta_{e}}\left(X\right)=\sigma (W_{e}X+b_{e}) \end{array}$$ (1)

Where $h\in {\text{R}}^{m}$(m<n) denotes the latent feature vector, σ(·) is a nonlinear activation function, and $W_{e}$ and $b_{e}$ are the encoder parameters. The decoder reconstructs the input as:


$$\begin{array}{c} \hat{X}=g_{\theta_{d}}\left(h\right)=\sigma (W_{d}h+b_{d}) \end{array}$$ (2)

Model training aims to minimize the reconstruction error between the input and its reconstruction. The Mean Squared Error (MSE) is commonly employed as the loss function:


$$\begin{array}{c} L\left(X,\hat{X}\right)=\frac{\text{1}}{N}\sum_{i=1}^{N}∥X_{i}-{\hat{X}}_{i}{∥}^{\text{2}} \end{array}$$ (3)

In anomaly detection, the autoencoder learns the latent distribution of normal data, resulting in low reconstruction errors for normal samples and higher reconstruction errors for anomalous samples. However, reconstruction error alone does not directly determine whether a sample should be classified as normal or abnormal. A decision threshold is required to convert continuous reconstruction errors into binary detection results. The threshold directly affects the balance between missed detections and false alarms. Therefore, this study uses Bayesian optimization to search for suitable threshold combinations for different reconstruction error metrics, which provides the basis for the threshold selection strategy described later.

### Long short-term memory

**Fig 2:**
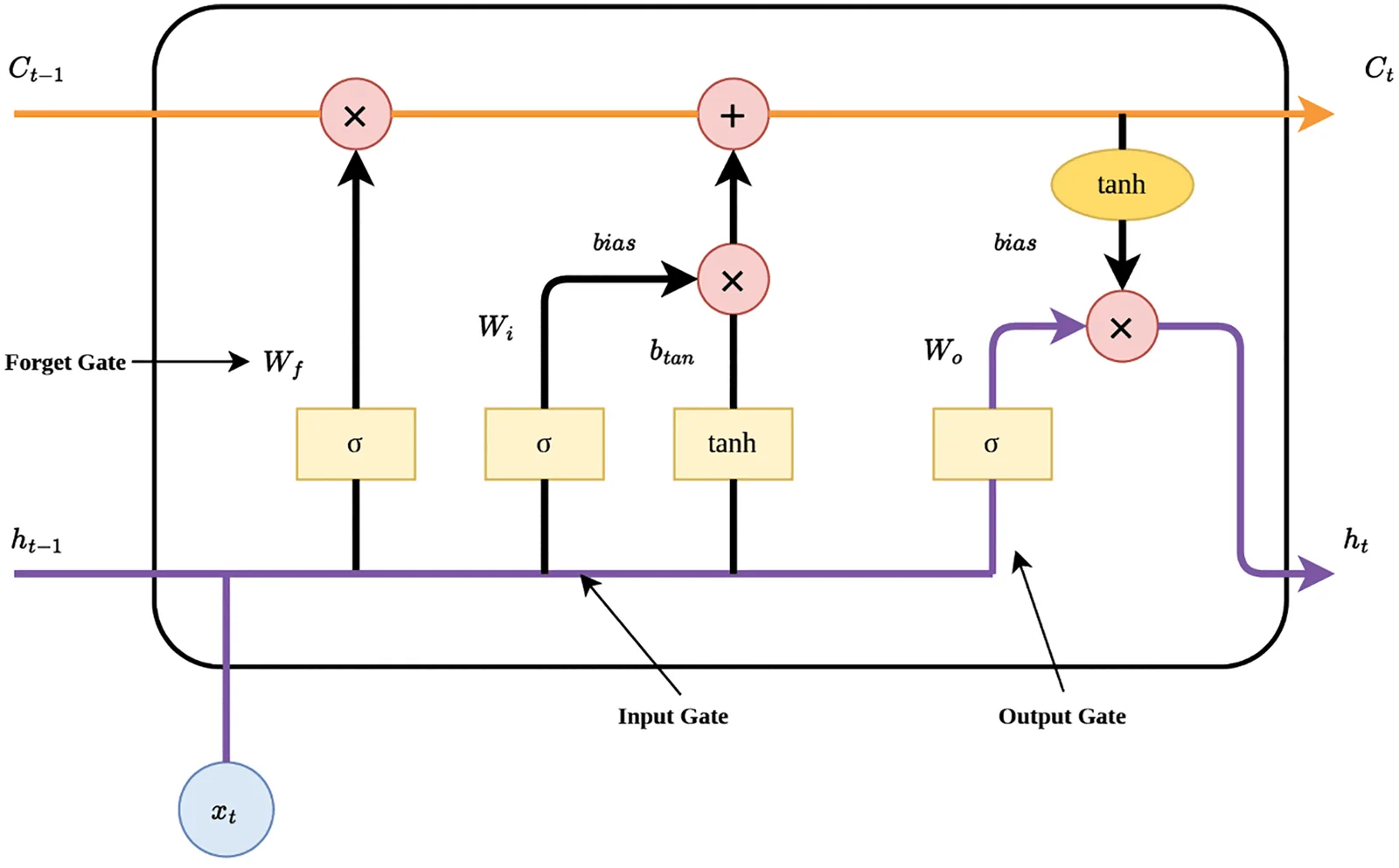
Structure of the LSTM unit.

Long Short-Term Memory is an advanced variant of Recurrent Neural Network (RNN) designed to address gradient vanishing and exploding problems in long sequence modeling [35]. By introducing gating mechanisms, LSTM enables effective learning of long-term temporal dependencies. An LSTM unit consists of a memory cell, a forget gate, an input gate, and an output gate, which collaboratively regulate information flow between the cell state and hidden state, as illustrated in Fig 2.

At time step t, the forget gate determines the retention of historical information based on the previous hidden state ${\text{h}}_{\text{t}-\text{1}}$ and the current input $x_{t}$:


$$\begin{array}{c} f_{t}=\sigma (W_{f}[H_{t-1},X_{t}]+b_{f}) \end{array}$$ (4)

The input gate controls the incorporation of new information through a candidate cell state:


$$\begin{array}{c} {\tilde{C}}_{t}=tanh(W_{c}[H_{t-1},X_{t}]+b_{c}) \end{array}$$ (5)

$$\begin{array}{c} i_{t}=\sigma (W_{i}[H_{t-1},X_{t}]+b_{i}) \end{array}$$ (6)

The cell state is then updated as:


$$\begin{array}{c} C_{t}=f_{t}⊙C_{t-1}+i_{t}⊙{\tilde{C}}_{t} \end{array}$$ (7)

Finally, the output gate generates the hidden state:


$$\begin{array}{c} o_{t}=\sigma (W_{o}[H_{t-1},X_{t}]+b_{o}) \end{array}$$ (8)

$$\begin{array}{c} H_{t}=o_{t}⊙tanh\left(C_{t}\right) \end{array}$$ (9)

Through the coordinated operation of these gates, LSTM achieves a balance between long-term memory retention and short-term feature updating.

### Multiple reconstruction error metrics

To quantify the discrepancy between the original input sequence and its reconstructed output, this study employs various reconstruction error metrics as anomaly scores. Different metrics capture different aspects of reconstruction quality, which may lead to significant differences in anomaly detection performance. In this study, six reconstruction error metrics are investigated, including Euclidean distance, Mean Absolute Error, Mean Squared Error, Root Mean Squared Error, Fast Dynamic Time Warping, and Cosine Similarity.

Let $X=\{x_{\text{1}},x_{\text{2}},...,x_{T}\}$ denote the original time series, and $\hat{X}=\{{\hat{x}}_{\text{1}},{\hat{x}}_{\text{2}},...,{\hat{x}}_{T}\}$denote the reconstructed sequence.

(1) Euclidean Distance

The Euclidean distance measures the overall geometric distance between two sequences:


$$\begin{array}{c} D_{EUC}\left(X,\hat{X}\right)=\sqrt{\sum_{t=1}^{T}(x_{t}-{\hat{x}}_{t}{)}^{\text{2}}} \end{array}$$ (10)

This metric is sensitive to large deviations and reflects the global reconstruction error.

(2) Mean Absolute Error

Mean Absolute Error (MAE) computes the average absolute difference between the original and reconstructed sequences:


$$\begin{array}{c} D_{\text{MAE}}\left(X,\hat{X}\right)=\frac{\text{1}}{T}\sum_{t=1}^{T}\left|x_{t}-{\hat{x}}_{t}\right| \end{array}$$ (11)

It is less sensitive to outliers compared to squared-error-based metrics.

(3) Mean Squared Error

MSE emphasizes larger deviations by squaring the error terms:


$$\begin{array}{c} D_{\text{MSE}}\left(X,\hat{X}\right)=\frac{\text{1}}{T}\sum_{t=1}^{T}(x_{t}-{\hat{x}}_{t}{)}^{\text{2}} \end{array}$$ (12)

This property makes it particularly sensitive to abrupt anomalies.

(4) Root Mean Squared Error

Root Mean Squared Error (RMSE) is the square root of MSE:


$$\begin{array}{c} D_{\text{RMSE}}\left(X,\hat{X}\right)=\sqrt{\frac{\text{1}}{T}\sum_{t=1}^{T}(x_{t}-{\hat{x}}_{t}{)}^{\text{2}}} \end{array}$$ (13)

It preserves the unit consistency with the original data while maintaining sensitivity to large errors.

(5) Fast Dynamic Time Warping

FastDTW is an efficient approximation of Dynamic Time Warping, which measures similarity between sequences with temporal misalignment:


$$\begin{array}{c} D_{\text{FastDTW}}\left(X,\hat{X}\right)={min}_{W}\sum_{\left(i,j\right)\in W}d\left(x_{i},{\hat{x}}_{j}\right) \end{array}$$ (14)

where W represents the optimal warping path. Unlike point-wise metrics, FastDTW allows nonlinear alignment in the time dimension, making it suitable for detecting temporal distortions and phase shifts in battery signals.

(6) Cosine Similarity

Cosine similarity evaluates the similarity in shape between two sequences:


$$\begin{array}{c} S_{\text{COS}}\left(X,\hat{X}\right)=\frac{X\cdot \hat{X}}{\|X\|\|\hat{X}\|} \end{array}$$ (15)

To maintain consistency with distance-based anomaly scoring, it can be transformed into a distance metric:


$$\begin{array}{c} D_{\text{COS}}\left(X,\hat{X}\right)=1-S_{\text{COS}}\left(X,\hat{X}\right) \end{array}$$ (16)

This metric is insensitive to magnitude differences and focuses on the directional similarity of sequences.

By integrating multiple reconstruction error metrics, this study aims to systematically evaluate their effectiveness and identify the most suitable metric for lead-acid battery anomaly detection. Due to the complex degradation patterns of lead-acid batteries, different metrics may exhibit varying sensitivity to gradual degradation and abrupt faults, which motivates a comparative investigation.

## Detection framework

### Anomaly detection framework

**Fig 3:**
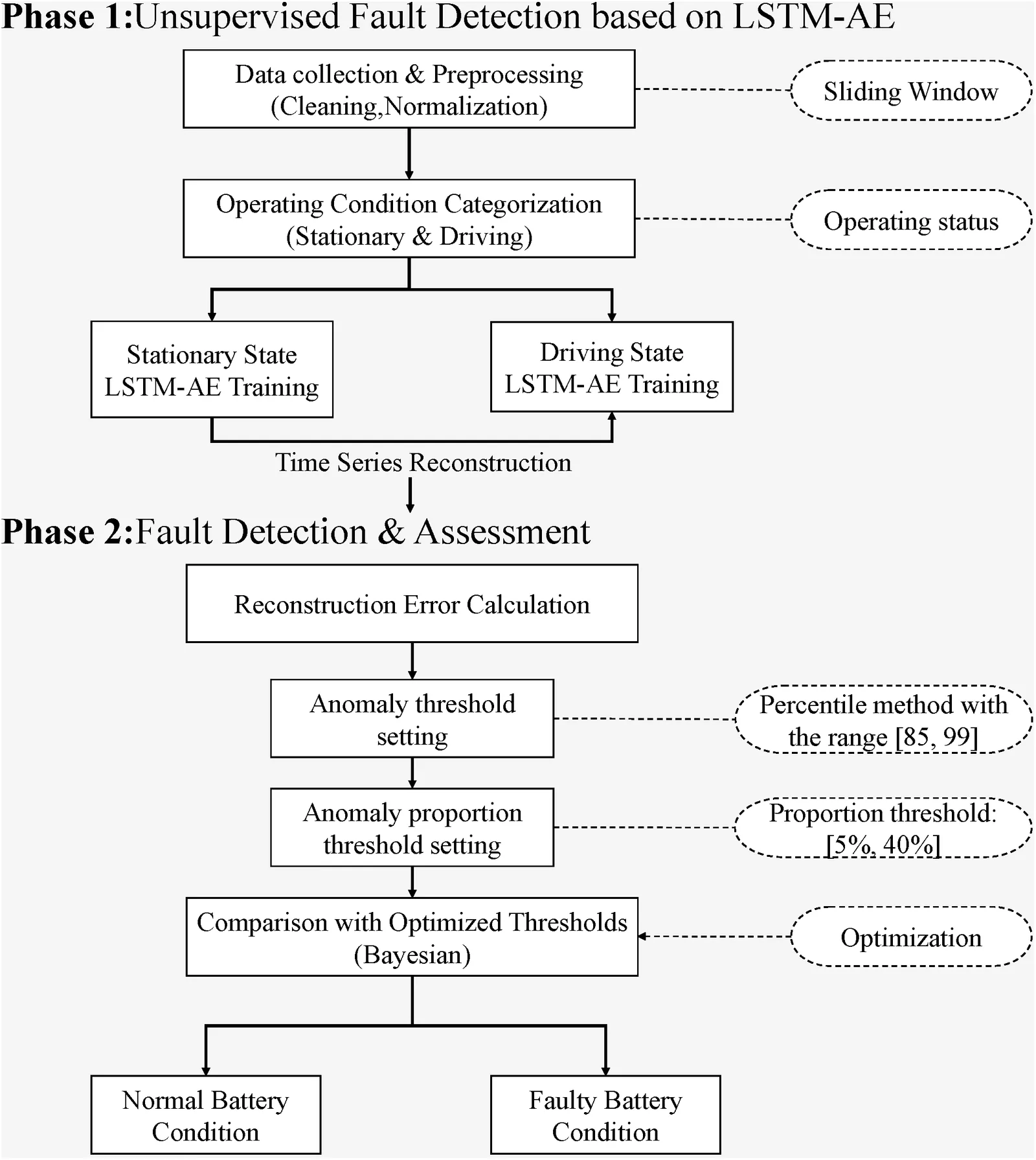
Framework of the proposed LSTM-AE fault detection.

The overall two-phase framework of the proposed unsupervised fault detection approach based on LSTM-AE, as shown in Fig 3.

Phase 1 begins with the collection and preprocessing of raw battery time-series data, including data cleaning and normalization. A sliding-window strategy is then applied to segment continuous voltage signals into fixed-length sequences. Based on vehicle operating status, the data are classified into two operating states: stationary and driving. To mitigate the effects of variability in operating conditions, two independent Long Short-Term Memory Autoencoders (LSTM-AEs) are trained separately for each state. Each model learns the normal temporal patterns of battery behavior and reconstructs the corresponding time-series data.

Phase 2 detects and assesses faults from the reconstruction results. We compute reconstruction errors with Euclidean distance, Mean Absolute Error, Mean Squared Error, Root Mean Squared Error, Fast Dynamic Time Warping, and Cosine Similarity. These metrics capture point-wise deviation, global magnitude differences, and temporal alignment. We perform all metric screening and threshold tuning on the validation set. For each metric, we initialize anomaly-detection thresholds with a percentile-based search over the 85th to 99th percentiles and evaluate anomaly-proportion thresholds from 5% to 40%. Bayesian optimization then selects the validation-optimal metric and its threshold parameters. After fixing the selected metric and thresholds, we apply them to the test set to report the final fault-detection result, classifying each battery condition as normal or faulty.

### Model architecture

**Fig 4:**
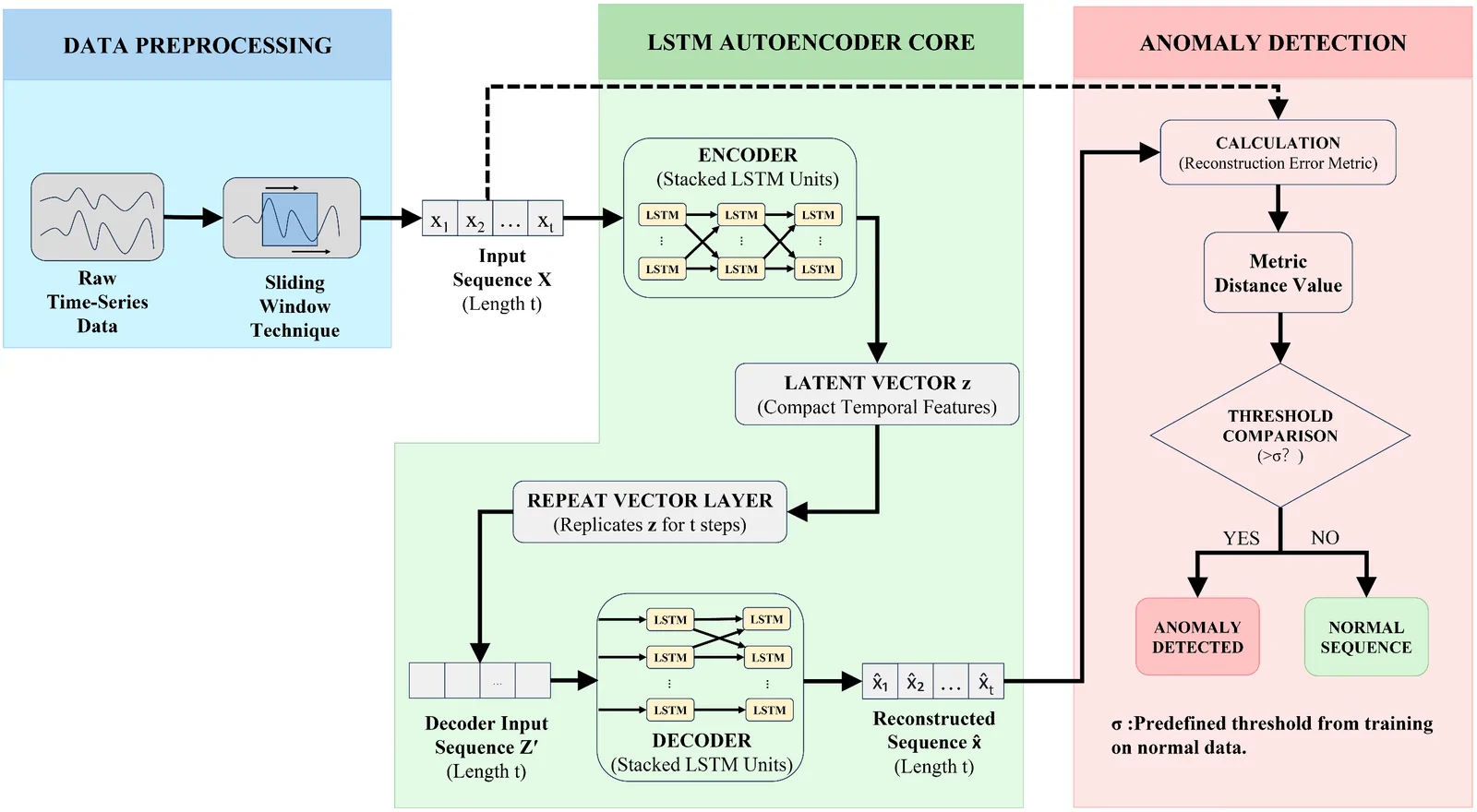
Architecture of the LSTM-AE model.

To capture the temporal features of battery time-series data and realize anomaly detection, this paper proposes an LSTM-AE model: LSTM is used to model temporal dependencies of the data, while AE is responsible for compressing and reconstructing the input. The specific architecture of the LSTM-AE model is illustrated in Fig 4.

The proposed LSTM-AE anomaly detection framework consists of an encoder, a latent representation layer, and a decoder. The framework evaluates reconstruction quality with multiple metrics that capture the discrepancy between the input sequence and its reconstruction. We perform metric screening and threshold selection only on the validation set: each candidate metric receives its own threshold, and we choose the metric that achieves the best validation performance. After fixing the selected metric and threshold, we use the test set only once to report the final anomaly-detection result.

Initially, a sliding window technique is applied to segment the time-series data into fixed-length sequences. Given an input sequence $\text{X}=({\text{x}}_{\text{1}},{\text{x}}_{\text{2}},\ldots ,{\text{x}}_{\text{t}})$ of length t, the encoder processes the sequence sequentially using stacked LSTM units and outputs the hidden state at the final time step as a compact latent representation. This latent vector z captures the global temporal features of the input sequence. A repeat vector layer then replicates zt times to generate a sequence$\;{\text{Z}}^{\prime }=(\text{R}{\text{V}}_{{\text{z}}_{\text{1}}},\ldots ,\text{R}{\text{V}}_{{\text{z}}_{\text{t}}})\in {\text{R}}^{\left\{\text{t}\times \text{d}\right\}}$,which matches the temporal length of the input and serves as the initial input to the decoder. The decoder, also composed of stacked LSTM units, takes sequence ${\text{Z}}^{\prime }$ and reconstructs the input as $\hat{\text{X}}=({\hat{\text{x}}}_{\text{1}},{\hat{\text{x}}}_{\text{2}},\ldots ,{\hat{\text{x}}}_{\text{t}})$. During training, the model is trained on normal data, enabling the network to learn typical temporal patterns of battery behavior. During inference, multiple reconstruction error metrics are calculated between X and $\hat{\text{X}}$, with each metric reflecting different aspects of reconstruction quality, such as amplitude deviation and temporal alignment. For each candidate metric, the anomaly detection threshold σ is determined using only the validation set. Candidate metrics are compared on the validation set, and the metric-threshold pair achieving optimal validation performance is fixed prior to evaluation. In the five-fold evaluation on the test set, this pre-determined metric and threshold are applied to all folds without repeated tuning. Each held-out test fold is utilized for performance assessment, and the final anomaly detection performance is obtained by aggregating results from the five test folds. Compared with single-metric methods, this validation-driven multi-metric selection strategy enhances adaptability and prevents test-set leakage.

## Experimental setup

### Dataset and preprocessing

The data were obtained from real-world historical vehicle records provided by a commercial vehicle manufacturer. The data were collected over three months and include operational data from 50 vehicles with normal battery performance and 27 with abnormal battery performance, sampled at one-minute intervals**.** The abnormal labels were provided by the manufacturer at the vehicle level based on its internal battery abnormality records. Specifically, each vehicle was labeled as having either normal or abnormal battery performance. The manufacturer did not provide fine-grained fault-type annotations, such as internal short circuit, capacity degradation, or open-circuit faults. Therefore, this study focuses on binary abnormal battery detection rather than fault-type diagnosis. The recorded variables include GPS time, battery voltage, and vehicle speed; battery voltage and vehicle speed were selected as input features for the model.

**Fig 5:**
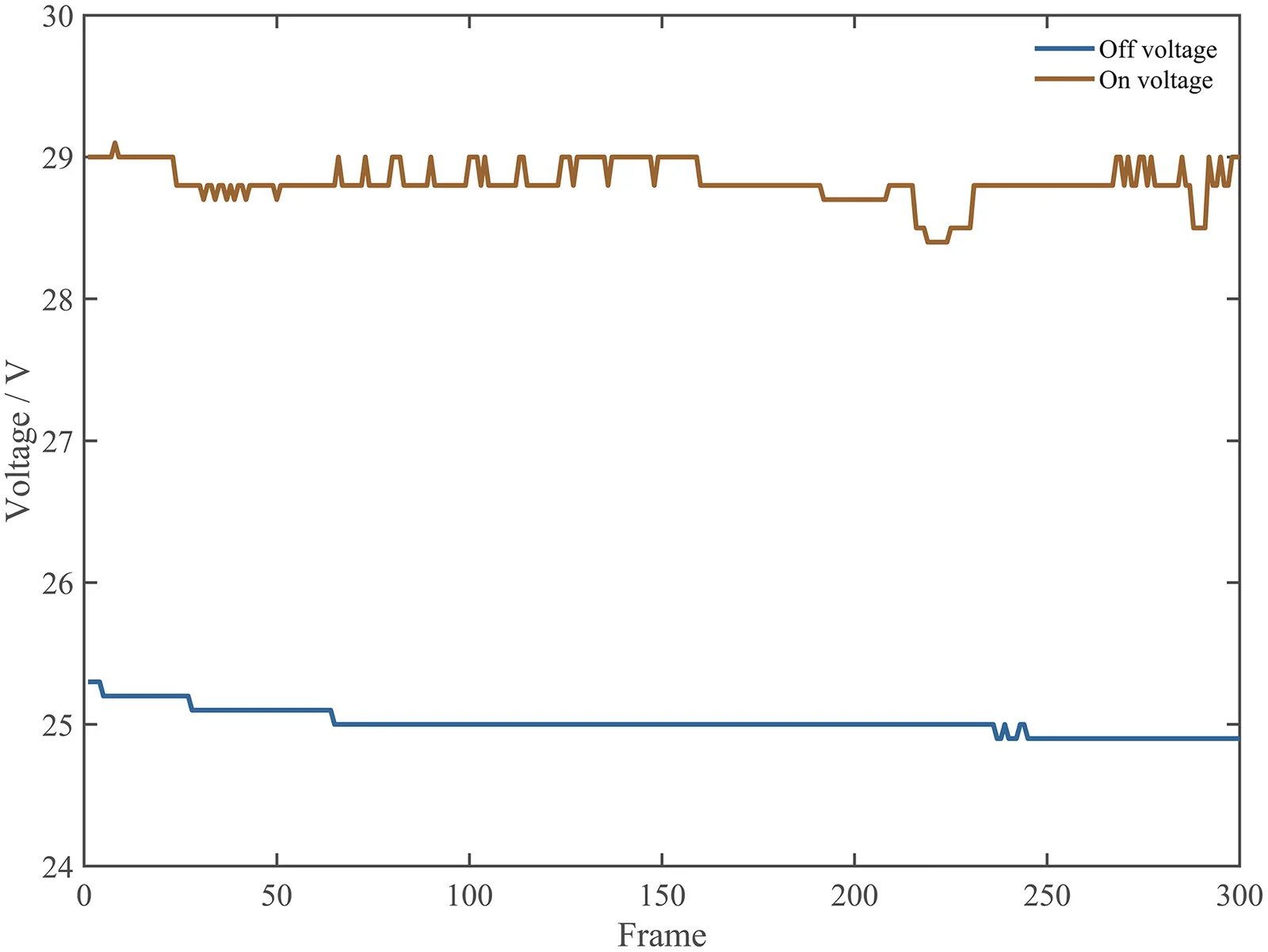
Voltage variation curves under different working conditions.


After the vehicle starts, the alternator charges the battery, raising the battery voltage to approximately 29 V; when the vehicle is stationary, the voltage remains around 25 V, as shown in Fig 5. The data are categorized into stationary and driving operating conditions, then cleaned, with missing values handled and outliers removed for each category. Sliding-window techniques [36] are employed to construct fixed-length time-series segments, ensuring temporal continuity. To eliminate the effects of differing feature scales and to accelerate model convergence, normalization is applied to the segmented sequences under both stationary and driving conditions, as defined below:

$$\begin{array}{c} q^{\prime }=\frac{q-q_{min}}{q_{max}-q_{min}} \end{array}$$ (17)

Where $q^{\prime }$denotes the normalized feature value, and $q_{\text{min}}\;$and $q_{\text{max}}\;$represent the minimum and maximum values of the feature. Given the scarcity of fault samples in real-world vehicle data and the difficulty in obtaining labels, the training set contains only normal data. This design follows the principle of unsupervised anomaly detection, which aims to learn normal behavioral patterns from normal samples without relying on labeled anomalous data. When test data deviates from these learned patterns, it can be identified as an anomaly. A random selection of 25 normal vehicles from the datasets was used as the training set for the unsupervised learning of the LSTM-AE model. The test set includes both normal and abnormal samples and is used solely for performance evaluation. It remains completely independent of the training and validation sets and is not used during model training or threshold selection. The detailed composition of the datasets is summarized in Table 1.

**Table 1:** Dataset construction details.

Datasets	Normal Vehicle	Normal Proportion	Abnormal Vehicle	Abnormal Proportion
Training Set	25	100%	0	0%
Validation set	10	50%	10	50%
Test Set	15	47%	17	53%

### Experimental equipment and hyperparameter settings

**Fig 6:**
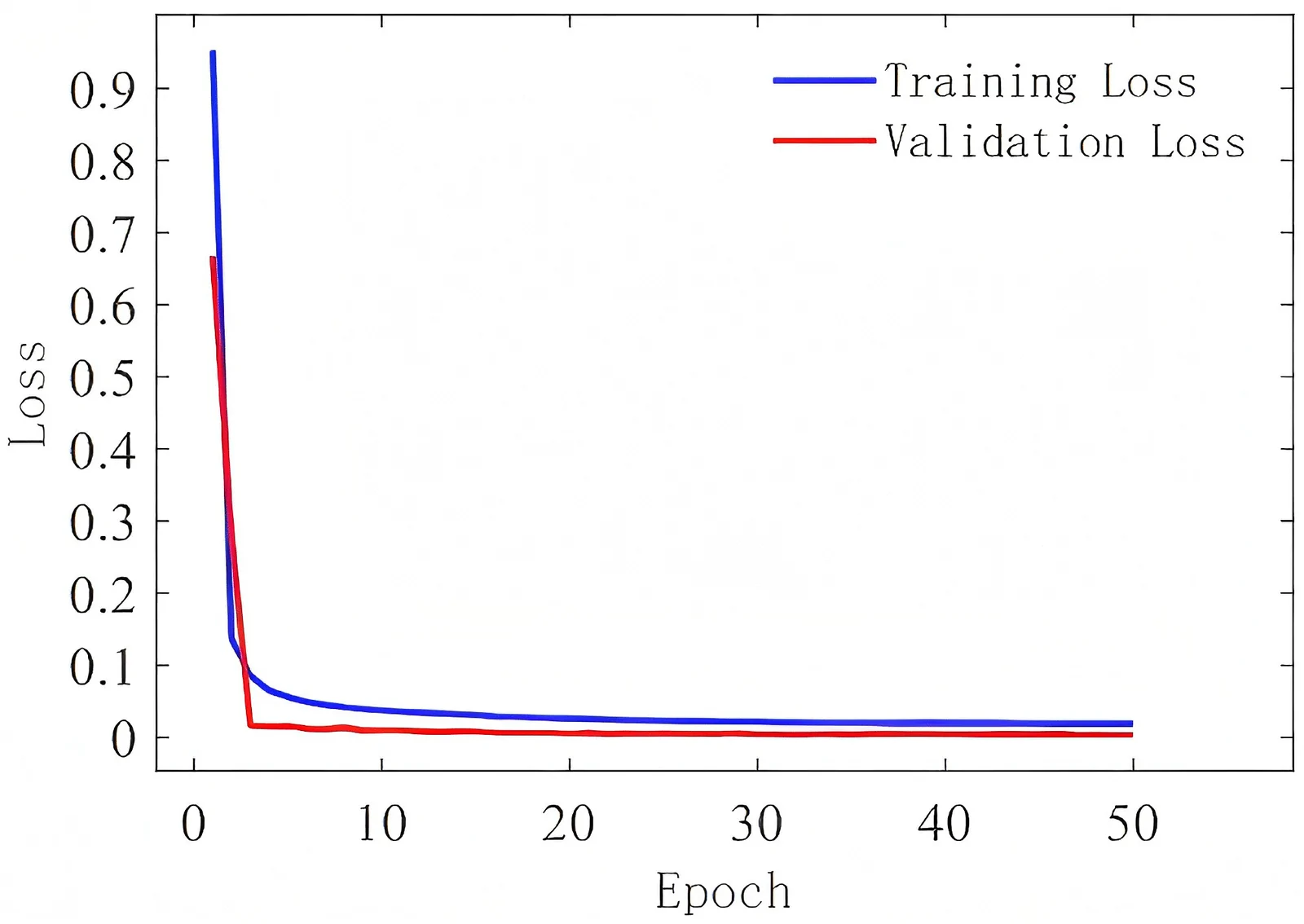
Variation of the loss function in driving condition model training.

**Fig 7:**
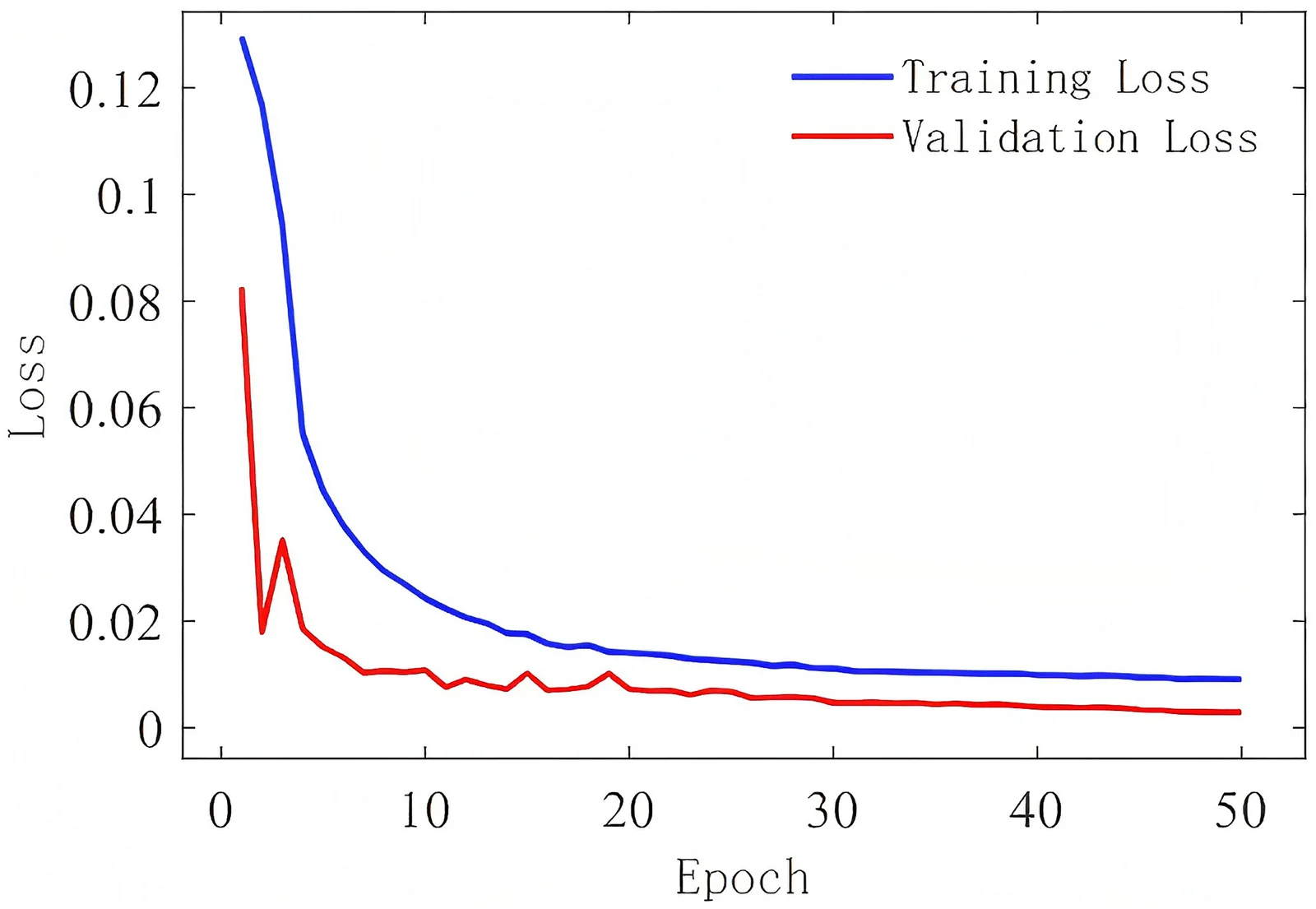
Variation of the loss function in static condition model training.

The hyperparameters of the proposed LSTM-AE model are configured as follows. The input and output sequence lengths are both set to 100. The LSTM hidden layer contains 64 neurons, and the latent space dimension is set to 50. Model training is performed using the Adam optimizer with an initial learning rate of 0.001. A stepwise learning rate decay strategy is applied, reducing the learning rate to 50% of its current value every 15 epochs. The model is trained for 50 epochs with a batch size of 32. Bayesian optimization selects thresholds on the validation set before testing. With these thresholds fixed, all comparative, reconstruction-metric, and ablation experiments use a five-fold evaluation on the held-out test set, and we report the results as mean ± standard deviation. All experiments were conducted on a workstation equipped with an Intel® Core™ i5-12400F CPU and an NVIDIA GeForce RTX 4060 Ti GPU. The evolution of the loss function during training is shown in Fig 6 and 7.

### Evaluation metrics

In the experimental section of this paper, accuracy, precision, recall, and F1-score are used as evaluation metrics for model performance, as shown in Equations (18)–(21).


$$\begin{array}{c} A=\frac{TP+TN}{TP+TN+FP+FN}\times 100\% \end{array}$$ (18)

$$\begin{array}{c} P=\frac{TP}{TP+FP}\times 100\% \end{array}$$ (19)

$$\begin{array}{c} R=\frac{TP}{TP+FN}\times 100\% \end{array}$$ (20)

$$\begin{array}{c} F1=2\times \frac{P\times R}{P+R}\times 100\% \end{array}$$ (21)

Where TP denotes the number of samples correctly predicted as anomalous, FP denotes the number of samples incorrectly predicted as anomalous, TN denotes the number of samples correctly predicted as normal, and FN denotes the number of samples incorrectly predicted as normal, as summarized in Table 2. Accuracy reflects the model’s overall discriminative ability to distinguish between normal and anomalous vehicle samples. Precision measures the reliability of the model’s positive (anomalous) predictions, while Recall evaluates its ability to identify actual anomalous samples correctly. The F1-score, defined as the harmonic mean of Precision and Recall, provides a balanced and comprehensive assessment of the model’s anomaly detection performance.

**Table 2:** Symbol definitions.

	Predicted Normal	Predicted Abnormal
Actual Normal	*TN*	*FP*
Actual Abnormal	*FN*	*TP*

### Strategy of threshold selection

Performance in the fault detection process is highly dependent on threshold parameters [37]. Traditional grid search methods require traversing all possible combinations of thresholds, which is inefficient and makes it difficult to search effectively within high-dimensional parameter spaces. Therefore, a Bayesian optimization framework [38] is introduced to optimize thresholds for multiple reconstruction error metrics, including Euclidean Distance, Mean Absolute Error, Mean Squared Error, Root Mean Squared Error, Fast Dynamic Time Warping, and Cosine similarity. Each reconstruction metric uses an independent threshold optimization process on the validation set. The percentile threshold is searched within the range of the 85th to 99th percentiles under both driving and stationary conditions. The abnormal segment ratio is set within the range of 5% to 40%, representing the minimum proportion of anomalous sequences required to classify a battery as faulty. The optimization objective is to maximize the F1-score on the validation set by jointly optimizing the percentile threshold and abnormality ratio for each reconstruction metric. The Bayesian optimization process is limited to a maximum of 30 iterations with a total runtime of no more than 1,800 seconds. After validation-set optimization, the selected thresholds are fixed and used in the subsequent five-fold evaluation, where test samples are excluded from threshold search. Tables 3–8 report the top candidate threshold combinations from the validation-set Bayesian optimization process rather than all explored candidates.

**Table 3:** Bayesian threshold optimization process for Euclidean.

Percentile	Abnormality Ratio	Static Threshold	Driving Threshold	F1 Score(%)
90	32%	0.065062	0.11511	68.7
99	10%	0.21976	0.17019	74.0
92	8%	0.071015	0.12016	76.2
96	9%	0.09099	0.13699	78.9
98	6%	0.12619	0.15371	81.1

**Table 4:** Bayesian threshold optimization process for MAE.

Percentile	Abnormality Ratio	Static Threshold	Driving Threshold	F1 Score(%)
98	38%	0.0048218	0.0070570	46.2
90	33%	0.0036281	0.0053010	63.0
93	8%	0.0040218	0.0058860	74.2
96	6%	0.0044218	0.0064720	78.2
99	5%	0.0052218	0.0076429	85.3

**Table 5:** Bayesian threshold optimization process for MSE.

Percentile	Abnormality Ratio	Static Threshold	Driving Threshold	F1 Score(%)
91	34%	0.00023033	0.00068988	66.6
97	23%	0.00054951	0.00010383	71.4
85	16%	0.00015481	0.00055973	74.4
94	11%	0.00030193	0.00080428	75.5
99	5%	0.00024148	0.00014482	79.7

**Table 6:** Bayesian threshold optimization process for RMSE.

Percentile	Abnormality Ratio	Static Threshold	Driving Threshold	F1 Score(%)
90	20%	0.0046006	0.0081398	70.2
85	5%	0.0039346	0.0074815	72.3
94	5%	0.0054948	0.0089682	75.5
95	8%	0.0058257	0.0093006	78.0
99	5%	0.015539	0.012034	79.7

**Table 7:** Bayesian threshold optimization process for FastDTW.

Percentile	Abnormality Ratio	Static Threshold	Driving Threshold	F1 Score(%)
98	39%	0.590824	0.854424	45.4
95	5%	0.481210	0.708626	82.0
86	7%	0.378410	0.555152	77.2
97	34%	0.541060	0.789951	64.0
99	5%	0.667528	0.964675	90.0

**Table 8:** Bayesian threshold optimization process for Cosine similarity.

Percentile	Abnormality Ratio	Static Threshold	Driving Threshold	F1 Score(%)
85	5%	0.00014782	0.00011724	72.3
90	5%	0.00025392	0.00014484	75.5
96	5%	0.00076056	0.0002236	84.2
97	5%	0.00010455	0.00026143	91.4
98	5%	0.00016395	0.00034569	94.7

As shown in Table 9, Bayesian optimization identifies different optimal threshold combinations for each reconstruction error metric on the validation set. The F1-scores in Tables 3–9 are validation-set scores used only for threshold selection, whereas the final detection performance is reported later through five-fold evaluation. The framework marks a sequence as anomalous if its reconstruction error exceeds the corresponding threshold under the given operating condition. Among all evaluated metrics, Cosine similarity achieves the best validation performance, with an F1-score of 94.7%, while FastDTW achieves an F1-score of 90.0%. These results indicate that similarity-based and alignment-aware metrics are more suitable for capturing temporal patterns in reconstructed battery sequences than point-wise error metrics.

**Table 9:** Validation-set optimal threshold combinations for each reconstruction metric.

Metric	Percentile	Abnormality Ratio	Static Threshold	Driving Threshold	F1 Score(%)
Euclidean	98	6%	0.12619	0.15371	81.1
MAE	99	5%	0.0052218	0.0076429	85.3
MSE	99	5%	0.00024148	0.00014482	79.7
RMSE	99	5%	0.015539	0.012034	79.7
FastDTW	99	5%	0.667528	0.964675	90.0
Cosine	98	5%	0.00016395	0.00034569	94.7

To further examine the sensitivity of detection performance to threshold selection, we analyzed the F1-score changes around the validation-set optimal threshold combinations. The candidate solutions near the optimum in Tables 3–8 show that small changes in percentile threshold and abnormality ratio generally lead to gradual rather than abrupt performance changes. For example, Cosine similarity achieves F1-scores of 91.4% and 94.7% at nearby threshold settings, while FastDTW achieves F1-scores of 82.0% and 90.0% near its optimal setting. These results indicate that the Bayesian optimization process selects a locally stable threshold region rather than an isolated threshold point. The final metric comparison is reported in the reconstruction performance analysis.

## Results and analysis

### Comparative experiments

With the thresholds selected on the validation set, we compared various anomaly detection methods using the same datasets and preprocessing methods under a five-fold evaluation on the held-out test set. The experiments selected anomaly detection methods mentioned in Reference [17], including Local Outlier Factor (LOF), K-Nearest Neighbor (KNN), Isolation Forest (IF), One-Class Support Vector Machine (OCSVM), and Variational Autoencoder (VAE), and compared them with the LSTM-AE-Cosine method.

As summarized in Table 10, classical methods show varying performance in battery anomaly detection. LOF achieves an F1-score of 79.9% ± 3.0%, but its precision indicates a relatively high false positive rate. KNN and VAE demonstrate moderate performance, with F1-scores of 81.9% ± 3.7% and 81.9% ± 3.1%, respectively. Isolation Forest and OCSVM show lower detection performance, with F1-scores of 59.7% ± 4.3% and 58.7% ± 3.6%, respectively.

**Table 10:** Results of comparative experiments.

Model	Accuracy(%)	Precision(%)	Recall(%)	F1 Score(%)
LOF	79.0 ± 2.6	78.6 ± 7.2	81.2 ± 4.9	79.9 ± 3.0
KNN	72.3 ± 7.9	71.0 ± 7.1	94.6 ± 3.2	81.9 ± 3.7
IForest	52.9 ± 3.1	65.2 ± 2.8	55.2 ± 6.7	59.7 ± 4.3
OCSVM	48.4 ± 6.2	60.1 ± 6.4	57.6 ± 2.6	58.7 ± 3.6
VAE	72.3 ± 6.6	71.1 ± 7.5	93.7 ± 5.2	81.9 ± 3.1
LSTM-AE-Cosine	91.3 ± 2.5	94.3 ± 3.6	94.7 ± 1.8	94.4 ± 1.5

In comparison, the LSTM-AE-Cosine method achieves higher values across all evaluation metrics, including 91.3% ± 2.5% accuracy, 94.3% ± 3.6% precision, 94.7% ± 1.8% recall, and 94.4% ± 1.5% F1-score. These results indicate improved anomaly detection performance under the given experimental conditions.

### Reconstruction performance analysis

The LSTM-AE model employs multiple reconstruction error metrics to evaluate reconstruction performance, including Euclidean distance, MAE, MSE, RMSE, FastDTW, and Cosine similarity. These metrics capture reconstruction quality from different perspectives, including point-wise differences, temporal alignment, and overall trend similarity. To validate the effectiveness of different reconstruction metrics, we compare their detection performance under the validation-selected thresholds. Table 11 reports the comparative results for the six reconstruction error metrics.

**Table 11:** Comparison of reconstruction metrics.

Method	Accuracy(%)	Precision(%)	Recall(%)	F1 Score(%)	Static Threshold	Driving Threshold
Euclidean	82.1 ± 7.5	80.5 ± 10.2	88.2 ± 5.8	83.6 ± 5.8	0.12619	0.15371
MAE	85.0 ± 5.1	90.8 ± 6.8	80.3 ± 10.5	84.2 ± 6.2	0.0052218	0.0076429
MSE	84.9 ± 6.1	85.9 ± 9.2	86.2 ± 6.7	85.6 ± 5.2	0.00024148	0.00014482
RMSE	82.0 ± 4.8	81.3 ± 5.6	84.3 ± 8.9	82.6 ± 5.3	0.015539	0.012034
FastDTW	88.7 ± 6.0	92.7 ± 5.1	89.4 ± 6.4	90.9 ± 4.7	0.667528	0.964675
Cosine	91.3 ± 2.5	94.3 ± 3.6	94.7 ± 1.8	94.4 ± 1.5	0.00016395	0.00034569

As shown in Table 11, Cosine achieves the best average detection performance after applying the validation-selected thresholds to the five-fold evaluation on the held-out test set.

As summarized in Table 11, Cosine similarity achieves the best detection performance, with an accuracy of 91.3% ± 2.5%, a precision of 94.3% ± 3.6%, a recall of 94.7% ± 1.8%, and an F1-score of 94.4% ± 1.5%. FastDTW also demonstrates strong performance, achieving an F1-score of 90.9% ± 4.7%. In comparison, traditional point-wise reconstruction error metrics show relatively lower performance. The Euclidean distance method achieves an F1-score of 83.6% ± 5.8%, while MAE, MSE, and RMSE obtain F1-scores of 84.2% ± 6.2%, 85.6% ± 5.2%, and 82.6% ± 5.3%, respectively. These results indicate that point-wise metrics are limited in capturing temporal dependencies and global sequence patterns.

Overall, similarity-based and alignment-aware metrics outperform conventional point-wise error metrics in this task. Cosine similarity achieves the best overall performance, indicating its strong capability in capturing global trend consistency between reconstructed and original sequences. FastDTW also shows competitive performance, particularly in handling temporal misalignment, making it a robust alternative for time-series anomaly detection. Consistent with the validation-set optimization results in Table 9, Cosine similarity was fixed as the primary reconstruction metric before test-set evaluation.

### Ablation experiments

The LSTM-AE model improves upon the standard autoencoder in two key aspects: (1) the incorporation of LSTM units to enhance temporal feature learning; (2) the adoption of cosine similarity as the reconstruction error metric. To systematically evaluate the individual contributions of these components to fault detection performance, an ablation study was conducted by progressively introducing these modifications. The results are presented in Table 12.

**Table 12:** Results of ablation experiments.

Model	Accuracy(%)	Precision(%)	Recall(%)	F1 Score(%)	Calculation Time(s)
AE	79.0 ± 2.6	78.6 ± 7.2	82.3 ± 10.1	80.1 ± 3.0	35.27 ± 2.3
AE-Cosine	84.0 ± 4.4	83.4 ± 4.3	86.2 ± 3.3	84.8 ± 3.8	21.38 ± 1.8
LSTM-AE	85.0 ± 5.1	90.8 ± 6.8	80.3 ± 10.5	84.2 ± 6.2	42.69 ± 5.6
LSTM-AE-Cosine	91.3 ± 2.5	94.3 ± 3.6	94.7 ± 1.8	94.4 ± 1.5	22.28 ± 3.2

As shown in Table 12, the baseline AE model, which relies on point-wise reconstruction error metrics, achieves relatively low detection performance, with an accuracy of 79.0% ± 2.6% and an F1-score of 80.1% ± 3.0%. This is mainly due to its limited ability to capture temporal dependencies in time-series data. By incorporating LSTM units, the LSTM-AE model, which also uses point-wise metrics, improves the accuracy and F1-score to 85.0% ± 5.1% and 84.2% ± 6.2%, respectively, demonstrating the advantage of temporal feature modeling. Replacing point-wise metrics with Cosine similarity further enhances performance. The AE-Cosine model achieves an F1-score of 84.8% ± 3.8% while reducing computation time to 21.38 ± 1.8 s, indicating that similarity-based metrics can better capture global trend consistency with lower computational cost. The LSTM-AE-Cosine model achieves the best overall performance, with an accuracy of 91.3% ± 2.5%, a precision of 94.3% ± 3.6%, and an F1-score of 94.4% ± 1.5%. Compared with the LSTM-AE model, it improves detection performance and reduces computation time from 42.69 ± 5.6 s to 22.28 ± 3.2 s.

Overall, the results indicate that both architectural enhancement and metric selection play critical roles in improving anomaly detection performance. The five-fold test evaluation results further show that the proposed LSTM-AE-Cosine model achieves a better balance between detection accuracy and computational efficiency, making it more suitable for practical fault detection applications.

## Discussion

The results show that, compared to classical unsupervised methods such as LOF, KNN, Isolation Forest, OCSVM, and VAE, the cosine similarity-based LSTM-AE framework proposed after comparing various reconstruction metrics demonstrates superior performance in lead-acid battery anomaly detection. This improvement is attributed to the joint effect of temporal modeling and similarity-based reconstruction evaluation. Specifically, the LSTM-based autoencoder captures long-term temporal dependencies in battery voltage sequences, while Cosine similarity measures the consistency of global trends between reconstructed and original signals, resulting in a more discriminative anomaly score. In addition, the operating-condition-aware training strategy reduces the influence of varying driving states and contributes to lower false alarm rates.

The comparison among reconstruction error metrics further shows that anomaly scoring plays a critical role in reconstruction-based detection. Point-wise metrics, such as MSE, MAE, RMSE, and Euclidean distance, measure numerical differences at corresponding time points. These metrics are sensitive to local voltage deviations, but they may also respond strongly to noise, short-term fluctuations, or slight temporal shifts in real vehicle data. In contrast, Cosine similarity evaluates the overall trend consistency between the original and reconstructed sequences. This makes it more suitable for detecting contextual or collective battery anomalies that appear as gradual changes in temporal patterns rather than isolated point deviations.

FastDTW also achieved competitive performance because it can compare sequences with temporal misalignment. This property is useful for vehicle battery data, where voltage responses may shift slightly under different driving states, charging behaviors, and load conditions. However, the results indicate that temporal alignment alone is not sufficient for the best anomaly discrimination in this task. FastDTW focuses on local alignment between reconstructed and original sequences, whereas Cosine similarity evaluates the consistency of the overall signal trend. For lead-acid battery faults that appear as gradual contextual or collective deviations, global trend consistency provides a more stable anomaly score than local time-warping alignment. Therefore, although FastDTW is useful for handling shifted time-series patterns, Cosine similarity better matches the anomaly characteristics of the commercial vehicle battery data used in this study.

From an application perspective, the proposed method is well suited for complex and non-stationary time-series scenarios, such as real-world vehicle operating conditions characterized by frequent state transitions, temporal misalignment, and noise. In such cases, methods based on static features or point-wise distance metrics may have limited ability to capture temporal dynamics, whereas the LSTM-AE-Cosine framework maintains more stable detection performance. Moreover, compared with alignment-based methods such as FastDTW, Cosine similarity is computationally simpler while preserving strong detection accuracy, making it more suitable for large-scale datasets and near real-time battery monitoring.

Several limitations should be noted. First, the abnormal vehicles in this dataset are labeled only at the vehicle level. The manufacturer provided binary abnormality labels but did not disclose detailed fault categories or maintenance-confirmed failure mechanisms. As a result, the proposed method detects manufacturer-labeled abnormal battery behavior, but it does not distinguish among specific fault types. Second, the model depends on sufficient high-quality normal data for training, and its performance may degrade when the dataset is limited or contains noise or mislabeled samples. Third, the method involves multiple hyperparameters, including anomaly detection thresholds. Although Bayesian optimization provides an effective approach for parameter selection, it introduces additional computational overhead. Finally, despite its improved efficiency, the overall framework may still face challenges in resource-constrained environments with strict real-time requirements.

Future work will focus on developing lightweight model architectures and adaptive thresholding strategies to further improve real-time performance and generalization. In addition, incorporating multi-source data, maintenance records, and diagnostic reports is expected to enhance detection accuracy, improve robustness, and support fine-grained fault classification.

## Conclusion

This paper presents a lead-acid battery anomaly detection method based on a Long Short-Term Memory autoencoder with multiple reconstruction error metrics. The approach is based on time-series reconstruction, where LSTM units replace the fully connected layers in a conventional autoencoder to better model sequential data. Multiple reconstruction metrics, including point-wise distance-based measures and similarity-based measures, are introduced to evaluate reconstruction quality from different perspectives. Bayesian optimization is applied to determine the optimal detection thresholds. The results indicate that incorporating LSTM units in both the encoder and decoder enables effective modeling of temporal dependencies in battery data. In addition, adopting similarity-based metrics improves the distinction between normal and abnormal sequences. Among all evaluated metrics, Cosine similarity achieves the best performance, demonstrating its effectiveness in capturing global trend consistency in time-series data. The proposed method relies only on time-series data collected during vehicle operation, and does not require labeled fault samples. Overall, the method provides a practical and efficient approach for anomaly detection in lead-acid batteries and shows potential for deployment in onboard monitoring systems and backend diagnostic platforms.

## Supporting information

S1 File Data and code. (ZIP)

## References

[1] Liu X, Teng T. Failure Causes and Effective Repair Methods of Lead-acid Battery. IOP Conf Ser: Earth Environ Sci. 2021;859(1):012083. doi: 10.1088/1755-1315/859/1/012083

[2] R A, Prasad A.N DrS. Prognostics and Health monitoring of Lead acid battery. ARAI J Mobi Tech. 2021;1(1):pp77-81. doi: 10.37285/ajmt.1.0.10

[3] Chandola V, Banerjee A, Kumar V. Anomaly detection: A survey. ACM Comput Surv. 2009;41(3):1–58. doi: 10.1145/1541880.1541882

[4] Xue Q, Li G, Zhang Y, Shen S, Chen Z, Liu Y. Fault diagnosis and abnormality detection of lithium-ion battery packs based on statistical distribution. Journal of Power Sources. 2021;482:228964. doi: 10.1016/j.jpowsour.2020.228964

[5] Yu-Hua Sun, Hurng-Liahng Jou, Jinn-Chang Wu. Diagnosis method for the degradation of lead-acid battery. In: 2009 IEEE International Symposium on Industrial Electronics, 2009. 1397–402. 10.1109/isie.2009.5222153

[6] Murari TB, Costa RCD, Pereira HBDB, Monteiro RL, Moret MA. Early detection of failing lead-acid automotive batteries using the detrended cross-correlation analysis coefficient. Appl Syst Innov. 2025;8(2):29. doi: 10.3390/asi8020029

[7] Hariprakash B, Martha SK, Jaikumar A, Shukla AK. On-line monitoring of lead–acid batteries by galvanostatic non-destructive technique. Journal of Power Sources. 2004;137(1):128–33. doi: 10.1016/j.jpowsour.2004.05.045

[8] Li X, Peng J, Li W, Song Z, Du X. Generative adversarial local density-based unsupervised anomaly detection. PLoS One. 2025;20(1):e0315721. doi: 10.1371/journal.pone.0315721 39854383 PMC11760566

[9] Qiu Y, Dong T, Lin D, Zhao B, Cao W, Jiang F. Fault diagnosis for lithium-ion battery energy storage systems based on local outlier factor. Journal of Energy Storage. 2022;55:105470. doi: 10.1016/j.est.2022.105470

[10] Haider SN, Zhao Q, Li X. Data driven battery anomaly detection based on shape based clustering for the data centers class. Journal of Energy Storage. 2020;29:101479. doi: 10.1016/j.est.2020.101479

[11] Shan F, Huang H, Liu X, Shen Z, Zeng J, Yu Z. Power battery voltage inconsistency fault identification method based on DBSCAN and dynamic K-value K-means++ joint clustering algorithm. Eng Res Express. 2025;7(3):035540. doi: 10.1088/2631-8695/adf59a

[12] Yang S, Wang X, Zhou S, Zhuang Y, Jin H, Chen J, et al. Multi-scenario failure diagnosis for lithium-ion battery based on coupling PSO-SA-DBSCAN algorithm. Journal of Energy Storage. 2024;99:113393. doi: 10.1016/j.est.2024.113393

[13] Jiang J, Li T, Chang C, Yang C, Liao L. Fault diagnosis method for lithium-ion batteries in electric vehicles based on isolated forest algorithm. Journal of Energy Storage. 2022;50:104177. doi: 10.1016/j.est.2022.104177

[14] Saxena S, Kang M, Xing Y, Pecht M. Anomaly Detection During Lithium-ion Battery Qualification Testing. In: 2018 IEEE International Conference on Prognostics and Health Management (ICPHM), 2018. 1–6. 10.1109/icphm.2018.8448735

[15] Zhang Z, Dong S, Li D, Liu P, Wang Z. Prediction and Diagnosis of Electric Vehicle Battery Fault Based on Abnormal Voltage: Using Decision Tree Algorithm Theories and Isolated Forest. Processes. 2024;12(1):136. doi: 10.3390/pr12010136

[16] Cheng X, Li X, Ma X. A method for battery fault diagnosis and early warning combining isolated forest algorithm and sliding window. Energy Science & Engineering. 2023;11(12):4493–504. doi: 10.1002/ese3.1593

[17] Goldstein M, Uchida S. A Comparative Evaluation of Unsupervised Anomaly Detection Algorithms for Multivariate Data. PLoS One. 2016;11(4):e0152173. doi: 10.1371/journal.pone.0152173 27093601 PMC4836738

[18] Dong S, Zhang B. Network traffic anomaly detection method based on deep features learning. J Electron Inf Technol. 2020;42(3):695–703. doi: 10.11999/JEIT190266

[19] Sakurada M, Yairi T. Anomaly detection using autoencoders with nonlinear dimensionality reduction. ACM Trans. 2014;8:4–11. doi: 10.1145/2689746.2689747

[20] Machlev R. EV battery fault diagnostics and prognostics using deep learning: Review, challenges & opportunities. Journal of Energy Storage. 2024;83:110614. doi: 10.1016/j.est.2024.110614

[21] Li P, Pei Y, Li J. A comprehensive survey on design and application of autoencoder in deep learning. Applied Soft Computing. 2023;138:110176. doi: 10.1016/j.asoc.2023.110176

[22] Wei Y, Jang-Jaccard J, Xu W, Sabrina F, Camtepe S, Boulic M. LSTM-Autoencoder-Based Anomaly Detection for Indoor Air Quality Time-Series Data. IEEE Sensors J. 2023;23(4):3787–800. doi: 10.1109/jsen.2022.3230361

[23] Lachekhab F, Benzaoui M, Tadjer SA, Bensmaine A, Hamma H. LSTM-Autoencoder Deep Learning Model for Anomaly Detection in Electric Motor. Energies. 2024;17(10):2340. doi: 10.3390/en17102340

[24] Kea K, Han Y, Kim T-K. Enhancing anomaly detection in distributed power systems using autoencoder-based federated learning. PLoS One. 2023;18(8):e0290337. doi: 10.1371/journal.pone.0290337 37594957 PMC10437833

[25] Andresini G, Appice A, Malerba D. Autoencoder-based deep metric learning for network intrusion detection. Inf Sci. 2021;569:706–27. doi: 10.1016/j.ins.2021.05.016

[26] Yao Y, Ma J, Feng S, Ye Y. SVD-AE: An asymmetric autoencoder with SVD regularization for multivariate time series anomaly detection. Neural Netw. 2024;170:535–47. doi: 10.1016/j.neunet.2023.11.023 38043373

[27] Goodfellow I, Pouget-Abadie J, Mirza M, Xu B, Warde-Farley D, Ozair S, et al. Generative adversarial networks. Commun ACM. 2020;63(11):139–44. doi: 10.1145/3422622

[28] Ozkat EC. Laser beam welding state classification: A deep learning framework for acoustic signal intelligence. Machines. 2026;14(6):652. doi: 10.3390/machines14060652

[29] Yilmaz A, Ozkat EC, Gul F. Signal Intelligence: Vibration-Driven Deep Learning for Anomaly Detection of Rotary-Wing UAVs. Drones. 2026;10(5):321. doi: 10.3390/drones10050321

[30] Altunkaya AN, Ozkat EC, Avci M. Analytical-to-AI pipeline: modeling and optimization of entropy generation in pulsating non-Newtonian heat flow. Comput Math Appl. 2026;205:195–211. doi: 10.1016/j.camwa.2025.12.021

[31] Lin J, He Y, Xu W, Guan J, Zhang J, Zhou S. Latent feature reconstruction for unsupervised anomaly detection. Appl Intell. 2023;53(20):23628–40. doi: 10.1007/s10489-023-04767-2

[32] Kwak BI, Han ML, Kim HK. Cosine similarity based anomaly detection methodology for the CAN bus. Expert Systems with Applications. 2021;166:114066. doi: 10.1016/j.eswa.2020.114066

[33] Gao Y, Yang Y, Ma Y, Xu W. Study on intelligent diagnosis of railway turnout switch based on improved FastDTW and time series segmentation under big data monitoring. Math Probl Eng. 2022;2022:7048813. doi: 10.1155/2022/7048813

[34] Pinaya WHL, Vieira S, Garcia-Dias R, Mechelli A. Autoencoders. Academic Press. 2020. p. 193–208. doi: 10.1016/B978-0-12-815739-8.00011-0

[35] Hochreiter S, Schmidhuber J. Long short-term memory. Neural Comput. 1997;9(8):1735–80. doi: 10.1162/neco.1997.9.8.1735 9377276

[36] Dai Q, Liu J, Yang JP. SWSEL: Sliding window-based selective ensemble learning for class-imbalance problems. Eng Appl Artif Intell. 2023;121:105959. doi: 10.1016/j.engappai.2023.105959

[37] Diallo AR, Homri L, Dantan J-Y. Reducing false alarms in fault detection: A comparative analysis between conformal prediction and classical methods applied to PCA and autoencoders. Journal of Process Control. 2025;152:103495. doi: 10.1016/j.jprocont.2025.103495

[38] Shahriari B, Swersky K, Wang Z, Adams RP, de Freitas N. Taking the Human Out of the Loop: A Review of Bayesian Optimization. Proc IEEE. 2016;104(1):148–75. doi: 10.1109/jproc.2015.2494218

